# Author Correction: Next-generation phenotyping integrated in a national framework for patients with ultrarare disorders improves genetic diagnostics and yields new molecular findings

**DOI:** 10.1038/s41588-025-02271-6

**Published:** 2025-06-24

**Authors:** Axel Schmidt, Magdalena Danyel, Kathrin Grundmann, Theresa Brunet, Hannah Klinkhammer, Tzung-Chien Hsieh, Hartmut Engels, Sophia Peters, Alexej Knaus, Shahida Moosa, Luisa Averdunk, Felix Boschann, Henrike Lisa Sczakiel, Sarina Schwartzmann, Martin Atta Mensah, Jean Tori Pantel, Manuel Holtgrewe, Annemarie Bösch, Claudia Weiß, Natalie Weinhold, Aude-Annick Suter, Corinna Stoltenburg, Julia Neugebauer, Tillmann Kallinich, Angela M. Kaindl, Susanne Holzhauer, Christoph Bührer, Philip Bufler, Uwe Kornak, Claus-Eric Ott, Markus Schülke, Hoa Huu Phuc Nguyen, Sabine Hoffjan, Corinna Grasemann, Tobias Rothoeft, Folke Brinkmann, Nora Matar, Sugirthan Sivalingam, Claudia Perne, Elisabeth Mangold, Martina Kreiss, Kirsten Cremer, Regina C. Betz, Martin Mücke, Lorenz Grigull, Thomas Klockgether, Isabel Spier, André Heimbach, Tim Bender, Fabian Brand, Christiane Stieber, Alexandra Marzena Morawiec, Pantelis Karakostas, Valentin S. Schäfer, Sarah Bernsen, Patrick Weydt, Sergio Castro-Gomez, Ahmad Aziz, Marcus Grobe-Einsler, Okka Kimmich, Xenia Kobeleva, Demet Önder, Hellen Lesmann, Sheetal Kumar, Pawel Tacik, Meghna Ahuja Bhasin, Pietro Incardona, Min Ae Lee-Kirsch, Reinhard Berner, Catharina Schuetz, Julia Körholz, Tanita Kretschmer, Nataliya Di Donato, Evelin Schröck, André Heinen, Ulrike Reuner, Amalia-Mihaela Hanßke, Frank J. Kaiser, Eva Manka, Martin Munteanu, Alma Kuechler, Kiewert Cordula, Raphael Hirtz, Elena Schlapakow, Christian Schlein, Jasmin Lisfeld, Christian Kubisch, Theresia Herget, Maja Hempel, Christina Weiler-Normann, Kurt Ullrich, Christoph Schramm, Cornelia Rudolph, Franziska Rillig, Maximilian Groffmann, Ania Muntau, Alexandra Tibelius, Eva M. C. Schwaibold, Christian P. Schaaf, Michal Zawada, Lilian Kaufmann, Katrin Hinderhofer, Pamela M. Okun, Urania Kotzaeridou, Georg F. Hoffmann, Daniela Choukair, Markus Bettendorf, Malte Spielmann, Annekatrin Ripke, Martje Pauly, Alexander Münchau, Katja Lohmann, Irina Hüning, Britta Hanker, Tobias Bäumer, Rebecca Herzog, Yorck Hellenbroich, Dominik S. Westphal, Tim Strom, Reka Kovacs, Korbinian M. Riedhammer, Katharina Mayerhanser, Elisabeth Graf, Melanie Brugger, Julia Hoefele, Konrad Oexle, Nazanin Mirza-Schreiber, Riccardo Berutti, Ulrich Schatz, Martin Krenn, Christine Makowski, Heike Weigand, Sebastian Schröder, Meino Rohlfs, Katharina Vill, Fabian Hauck, Ingo Borggraefe, Wolfgang Müller-Felber, Ingo Kurth, Miriam Elbracht, Cordula Knopp, Matthias Begemann, Florian Kraft, Johannes R. Lemke, Julia Hentschel, Konrad Platzer, Vincent Strehlow, Rami Abou Jamra, Martin Kehrer, German Demidov, Stefanie Beck-Wödl, Holm Graessner, Marc Sturm, Lena Zeltner, Ludger J. Schöls, Janine Magg, Andrea Bevot, Christiane Kehrer, Nadja Kaiser, Ernest Turro, Denise Horn, Annette Grüters-Kieslich, Christoph Klein, Stefan Mundlos, Markus Nöthen, Olaf Riess, Thomas Meitinger, Heiko Krude, Peter M. Krawitz, Tobias Haack, Nadja Ehmke, Matias Wagner

**Affiliations:** 1 Institute of Human Genetics, University of Bonn, Medical Faculty and University Hospital Bonn, Bonn, Germany; 2 Institute for Medical Genetics and Human Genetics, Charité – Universitätsmedizin Berlin, Berlin, Germany; 3 BIH Charité Clinician Scientist Program, Berlin Institute of Health at Charité – Universitätsmedizin Berlin, Berlin, Germany; 4 Institute for Medical Genetics and Applied Genomics, University of Tübingen, Tübingen, Germany; 5 Institute of Human Genetics, Klinikum rechts der Isar, School of Medicine, Technical University of Munich, München, Germany; 6 Institute for Genomic Statistics and Bioinformatics, University of Bonn, Medical Faculty and University Hospital Bonn, Bonn, Germany; 7 Institut für Medizinische Biometrie, Informatik und Epidemiologie, University of Bonn, Medical Faculty and University Hospital Bonn, Bonn, Germany; 8 Institute for Medical Genetics, Stellenbosch University, Cape Town, South Africa; 9 Department of Pediatrics, University Hospital Düsseldorf, Düsseldorf, Germany; 10 Institute for Human Genetics and Genomic Medicine, Medical Faculty, Uniklinik RWTH Aachen University, Aachen, Germany; 11 Core Uni Bioinformatics, Berlin Institute of Health at Charité – Universitätsmedizin Berlin, Berlin, Germany; 12 Department of Pediatrics, Charité – Universitätsmedizin Berlin, Berlin, Germany; 13 Department of Pediatric Neurology, Charité – Universitätsmedizin Berlin, Berlin, Germany; 14 Center for Chronically Sick Children, Charité – Universitätsmedizin Berlin, Berlin, Germany; 15 Institute of Cell and Neurobiology, Charité – Universitätsmedizin Berlin, Berlin, Germany; 16 Department of Human Genetics, Ruhr University Bochum, Bochum, Germany; 17 Department of Pediatrics Bochum and CeSER, Ruhr University Bochum, Bochum, Germany; 18 Center for Rare Diseases, University of Bonn, Medical Faculty and University Hospital Bonn, Bonn, Germany; 19 Department of Neurology, University of Bonn, Medical Faculty and University Hospital Bonn, Bonn, Germany; 20 Clinic for Internal Medicine III, University of Bonn, Medical Faculty and University Hospital Bonn, Bonn, Germany; 21 University Center for Rare Diseases, University Hospital Carl Gustav Carus, Dresden, Germany; 22 Department of Pediatrics, University Hospital Carl Gustav Carus, Dresden, Germany; 23 Institute for Clinical Genetics, University Hospital Carl Gustav Carus, Dresden, Germany; 24 Department of Neurology, University Hospital Carl Gustav Carus, Dresden, Germany; 25 Institute of Human Genetics, University Hospital Essen, Essen, Germany; 26 Department of Pediatrics II, University Hospital Essen, Essen, Germany; 27 Department of Neurology, University Hospital Halle, Halle, Germany; 28 Institute of Human Genetics, University Hospital Hamburg-Eppendorf, Hamburg, Germany; 29 Martin Zeitz Center for Rare Diseases, University Hospital Hamburg-Eppendorf, Hamburg, Germany; 30 Institute of Human Genetics, Heidelberg University, Heidelberg, Germany; 31 I. Department of Medicine, University Hospital Hamburg-Eppendorf, Hamburg, Germany; 32 Department of Pediatrics, University Hospital Hamburg-Eppendorf, Hamburg, Germany; 33 Center for Child and Adolescent Medicine, University Hospital Heidelberg, Heidelberg, Germany; 34 Institute of Human Genetics, University Hospital Schleswig-Holstein, Lübeck, Germany; 35 Center for Rare Diseases, University Hospital Schleswig-Holstein, Lübeck, Germany; 36 Department of Neurology, University Hospital Schleswig-Holstein, Lübeck, Germany; 37 Institute for Neurogenetics, University Hospital Schleswig-Holstein, Lübeck, Germany; 38 Institute of Systems Motor Science, University of Lübeck, Lübeck, Germany; 39 Institute of Neurogenetics, University of Lübeck, Lübeck, Germany; 40 Institute of Human Genetics, University of Lübeck, Lübeck, Germany; 41 Department of Human Genetics, University Hospital Schleswig-Holstein, Lübeck, Germany; 42 Department of Nephrology, Klinikum rechts der Isar, School of Medicine, Technical University of Munich, München, Germany; 43 Institute of Neurogenomics, Helmholtz Zentrum München, München, Germany; 44 Department of Neurology, Medical University of Vienna, Wien, Austria; 45 Department of Paediatrics, Adolescent Medicine and Neonatology, München, Germany; 46 Dr. von Hauner Children’s Hospital, University Hospital Munich, München, Germany; 47 Institute of Human Genetics, University of Leipzig Medical Center, Leipzig, Germany; 48 Center for Rare Diseases, University of Leipzig Medical Center, Leipzig, Germany; 49 Center for Rare Diseases, University of Tübingen, Tübingen, Germany; 50 Department of Neurology, University of Tübingen, Tübingen, Germany; 51 Department of Pediatric Neurology and Developmental Medicine, University of Tübingen, Tübingen, Germany; 52 Department of Genetics and Genomic Sciences, Icahn School of Medicine at Mount Sinai, New York, NY USA; 53 Berlin Centre for Rare Diseases, Charité – Universitätsmedizin Berlin, Berlin, Germany

**Keywords:** Genetics research, Genetic testing

Correction to: *Nature Genetics* 10.1038/s41588-024-01836-1, published online 22 July 2024.

In the version of the article initially published, the surname of Meghna Ahuja Bhasin appeared incorrectly (as Basin) and has now been amended in the HTML and PDF versions of the article.

