# Supplementary material for: Next-generation phenotyping integrated in a national framework for patients with ultrarare disorders improves genetic diagnostics and yields new molecular findings
Source: Nat Genet. 2024 Jul 22;56(8):1644–53. doi: 10.1038/s41588-024-01836-1 (PMC11319204; doi:10.1038/s41588-024-01836-1)
Supplement: Supplementary file 1 — Supplementary note and Figs. 1–14 and legends for Supplementary Tables 1–7. [file 41588_2024_1836_MOESM1_ESM.pdf]

nature genetics

Supplementary information https://doi.org/10.1038/s41588-024-01836-1

Next-generation phenotyping integrated in a national framework for patients with ultrarare disorders improves genetic diagnostics and yields new molecular findings

In the format provided by the authors and unedited

Contents

Supplementary Note 2
- Novel disease candidate genes 2
- Gene length calculation 3
- Parental Mosaicism 3
- Secondary Findings 4
- Case reports of particular interest 5
- GestaltMatcher integration with other tools 8

Supplementary Figures 9
Supplementary Tables 21
References 22

Supplementary Note

Novel disease candidate genes

A total of 57 different candidate genes in 65 affected individuals from 63 unrelated families had an evidence score of ≥1 (Supplementary Data Table 3). The inheritance pattern was autosomal dominant for 41 genes (evidence score range 1-7); autosomal recessive (AR) for 15 genes (evidence score range 1-5); and X-chromosomal for one gene (evidence score 1). Sixteen genes have subsequently acquired diagnostic-grade gene (DGG) status. Selected cases are described below, most of which have been described in detail in previous publications.

SMARCA5

In *SMARCA5*, a gene that encodes a chromatin remodeler, we identified *de novo* variants in two patients with neurodevelopmental delay and similar dysmorphic features. The phenotype-gene association was strengthened by the identification of 10 additional cases from other cohorts. Rescue experiments with wildtype transcripts in Drosophila suggested that the identified *de novo* variants had a hypomorphic effect[1].

KCNN2

In another individual with learning disabilities, autism, dystonia, and intention tremor, we identified a *de novo* missense variant in *KCNN2.* This gene encodes a small-conductance calcium-activated potassium channel protein. Interestingly, a preexisting rat model with a missense substitution identical to that found in the affected individual partially mirrors this phenotype, with abnormal locomotor activity and tremors. The identification of nine additional individuals from other cohorts, and the results of functional analyses of the variants with respect to channel function, established *KCNN2* as a dominant disease-associated gene for a neurodevelopmental movement disorder[2].

MAPKAPK5

We delineated a recognizable syndrome with multi-organ manifestations for biallelic truncating variants in *MAPKAPK5* (MAPK-activated protein kinase 5). Patients presented with severe developmental delay, variable brain anomalies, congenital heart defects, facial dysmorphism, and a distinct type of synpolydactyly involving the presence of an additional hypoplastic digit between the fourth and fifth digits of the hands and/or feet[3].

OAS1

We established the gene *OAS1*, which encodes a type I interferon–induced, intracellular double-stranded RNA (dsRNA) sensor that is required for antiviral defense, as a DGG. A *de novo* gain-of-function (GOF) variant that caused dsRNA-independent OAS1 activity was identified in a patient with an autoinflammatory immunodeficiency. The latter was characterized by B-cell and monocyte apoptosis-related hypogammaglobulinemia and pulmonary alveolar proteinosis. Via an international network of experts on inborn errors of immunity, we identified three additional *de novo OAS1* GOF variants in a total of six patients, and demonstrated that allogeneic hematopoietic cell transplantation can be applied as a curative approach.

Gene length calculation

Coding sequence length was calculated on the basis of Consensus Coding Sequences (CCDS; CCDS.current.txt file downloaded on 22 July 2021 from https://ftp.ncbi.nih.gov/pub/CCDS/current_human/). A list of genes that cause Mendelian disease according to Online Mendelian Inheritance in Man database (OMIM) (n=4,369) was obtained using the files genemap2.txt and mimTitles.txt, as downloaded on 15 July 2021. Note that a minority of genes of TRANSLATE-NAMSE and OMIM was not represented in the Consensus Coding Sequence files used (e.g. *SHANK3*). Supplementary Figure 9 displays boxplots comparing: all genes (n=18,598); OMIM disease-causing genes (n=4,369); TRANSLATE NAMSE ES cohort disease-associated genes (n=330); and TRANSLATE NAMSE exome sequencing cohort research genes (n=24). For known disease-associated genes in the TRANSLATE NAMSE exome sequencing cohort (TN_Diagnostic), and genes for which a novel disease association was found (TN_Research), the mean coding length was significantly longer than for all genes or for all OMIM genes.

Parental Mosaicism

Amongst the 228 solved cases that were attributable to *de novo* variants, three probable or certain parental mosaics affecting at least the germ cells were detected. This was achieved via visual inspection of all parental sequence reads in Integrative Genomics Viewer (IGV) for the presence of the corresponding variant reads (≥ 1). In two of the three families, the unequivocal presence of low-level parental mosaicism was not confirmed, since in family 2, this was only hinted at by a single parental read, while in family 3 no parental mosaicism was demonstrated. However, parental mosaicism is the most probable explanation for recurrence of the “de novo” variant in a second child.

In family 1, which had only one affected member (case 432), a pathogenic, heterozygous nonsense variant in *ASXL3* was detected, leading to the assignment of a diagnosis of autosomal dominant Bainbridge-Ropers-Syndrome (OMIM #615485). The variant was also detected in two out of 69 exome sequencing reads from the mother and one out of 60 reads from the father. For the paternal sequence reads, low-quality scores pointed to an artifact. In addition, three unrelated individuals from the same sequencing run carried the variant on one read respectively, suggesting batch contamination. Sanger sequencing revealed the low intensity presence of the variant in maternal lymphocyte DNA, while no evidence for the variant was detected in paternal DNA. Since the latter did not rule out the presence of low-level paternal mosaicism, both parents were sequenced again at a higher coverage. Here, the presence of the variant, and at high quality, was found in 10 out of 177 maternal reads, while no such variant was detected in 197 paternal reads.

In family 2, which included two siblings with intellectual disability (case 435 and his brother), a heterozygous, likely pathogenic variant in *FOXG1* (*FOXG1* syndrome, OMIM #613454) was detected in both individuals. DNA from maternal blood was subjected to exome sequencing, and the variant was found in one of 132 reads. No evidence of contamination was found. Sanger sequencing of DNA from both a second maternal blood sample and a maternal buccal swab was then performed. The variant was not detected in either sample.

In family 3, parental mosaicism was established on the basis of the presence of a heterozygous, likely pathogenic variant in the gene *PUF60,* which explained the observed developmental delay in two brothers (cases 443 and 444; Verheij syndrome, OMIM #615583). DNA from the blood of both parents was then subjected to exome sequencing.

The variant was not detected in either sample, despite a high coverage of 226 and 201 sequencing reads, respectively.

These examples illustrate the frequency of low-level parental mosaicism and its relevance in terms of counseling for recurrence risk. Notably, only two of these three parental mosaics would have been detected in parental next generation sequencing (NGS) reads if the criteria proposed by Gambin and colleagues had been applied, according to which only variants that are present in at least two or more reads and that are absent in reference databases are classified as candidate low-level mosaic single nucleotide variants[4]. The findings in family 3 again demonstrate that the apparent absence of a variant - even in high-coverage parental NGS data – does not exclude gonadal mosaicism.

The detection in the exome sequencing analysis of three probable or proven parental mosaics among 228 patients would correspond to a frequency of 1.3%. This is in agreement with two previous studies, which investigated the frequency of low-level parental mosaicism for pathogenic or likely pathogenic variants identified in affected children. Wright and colleagues analyzed 4,293 exome sequencing trios and identified and validated eight low-level parental mosaics, six of which were considered likely pathogenic or pathogenic (corresponding to xxx% of the trios)[5]. Cao and colleagues analyzed a cohort of approximately 12,000 samples submitted for clinical exome sequencing and identified 0.3% parental mosaics, via analysis of parental NGS reads or via Sanger sequencing of parental DNA[6].

Our findings highlight the importance of the visual inspection of parental NGS data for low-level mosaicism. In addition, the analyses emphasize the importance of recognizing pedigree constellations that are suggestive of parental gonadal mosaicism.

Secondary Findings

Pathogenic (class 5) and likely pathogenic (class 4) variants were only reported to the respective patient if the individual or their legal guardian had previously consented to being informed about secondary findings (SF). The list of actionable genes was based on the recommendations of the American College of Medical Genetics (ACMG, v2.0)[7,8]. However, variants in seven additional genes were reported to patients following discussions within the respective multidisciplinary teams, since it was concluded that these variants fulfilled the criteria for actionable findings (see below). A list of all SFs identified in the TRANSLATE NAMSE exome sequencing cohort is provided in Supplementary Table 7.

Medically actionable SFs that were found in genes unrelated to the phenotype, but which had immediate implications for management, were identified in a total of 17 patients (1.1%). In eight cases, the SFs concerned genes included in the ACMG list v2.0[8]. Risk management counseling was provided to the affected individuals, in accordance with the NCCN Guidelines[9]. For the remaining cases, six had variants in the cancer predisposition genes *DICER1, PALB2, CHEK2, BRIP1, SBDS,* and *BAP1,* respectively. In view of current recommendations that mutation carriers should avoid exposure to specific agents[10], two individuals were informed of the SF of X-linked glucose-6-phosphate dehydrogenase deficiency (G6PD; OMIM #300908). Similarly, the reporting of the SF of a pathogenic variant in both *KCNJ11* and in *GHRHR* was of direct clinical benefit to the respective patients and their families, due to the availability of clinical enzyme therapy for maturity onset diabetes of the young type 13 (MODY13, OMIM #616329) and isolated growth hormone deficiency type 4 (OMIM #618157) respectively. Among the ACMG listed genes, the only recurrent findings concerned variants in *BRCA2* and *LDLR,* which were detected in three patients respectively.

In the present multi-center study, the identification and reporting of SF was non-standardized due to cross-center differences in procedures and policies. In particular, cross-center

differences were evident with regards to trio exome sequencing, when variants were identified that predisposed to a disorder with an onset in adulthood. Here, some centers returned medically actionable results to the adult proband only and did not automatically inform the respective parents. Centers also differed in terms of both their lists of actionable genes and if they reported heterozygous variants in genes with autosomal recessive inheritance patterns. For some conditions, variants of uncertain significance (VUS) were also reported if a simple clinical test was available for further clarification. One such case involved the identification of a VUS in a gene associated with long QT syndrome, which prompted a recommendation for an electrocardiogram. The number of patients with pathogenic variants in genes from the ACMG list was lower than in previous exome sequencing and genome sequencing (GS) studies, which found SFs in 1.2% to 11% of cases[11–15]. This high cross-study variability in SF rates is attributable to: 1) the high discrepancy in terms of the genetic diagnoses that were considered medically actionable; 2) the informed consent procedure (opt-out for non-ACMG SF versus non-opt-out; 3) the applied classification strategy; and 4) the filtering procedures used for variants during exome analysis. This underlines the need for an updated and curated set of actionable genes, as well as updated standards for analysis procedures, the determination of the pathogenicity of variants, and guidelines for returning results to the patient.

Case reports of particular interest

KANSL1 (next-generation phenotyping)

In a female individual (case 393), who first presented at the age of eight years with cognitive impairment, generalized muscle weakness, nevi, iron deficiency anemia, poor fine motor coordination, recurrent respiratory infections, and dysmorphic facies, facial image analysis suggested a high syndromic similarity to Koolen-De-Vries syndrome (Supplementary Figure 5). After inconclusive exome sequencing, targeted analyses of *KANSL1* and GS were performed, which revealed a 4.7 kb *de novo* deletion in *KANSL1,* NM_001193466:c.1849-4661_1895del. This example illustrates the value of high gestalt scores, which can be used as an indicator of the need for a targeted search for non-coding disease-causing mutations in the specific genomic region that is associated with the phenotype.[16]

BICD2 and *TANGO2* (dual diagnosis)

A 13-year-old boy (case 144) with non-consanguineous parents presented with global developmental delay, muscular hypotonia, bilateral optic atrophy, cardiac arrhythmia, hypothyroidism, chronic urticaria, and two episodes of rhabdomyolysis. After an uneventful pregnancy, full term delivery, and normal postnatal development, he had experienced his first atonic seizure at the age of one year. Following bilateral mastoiditis and subsequent mastoidectomy and paracentesis, a first episode of rhabdomyolysis occurred at the age of 16 months, and this later recurred in the context of febrile infections. His development was delayed, with the onset of unassisted walking and first words at the age of 5 years. At the age of 11 years, he was able to speak around 30 words and use 2-word sentences. At 18 months, he experienced an unexplained loss of consciousness in association with gastroenteritis. At 25 months, he developed an intermittent loss of muscle tone, with a fixed gaze, ptosis, and hypersalivation consistent with atonic seizures. At the age of 13 years, the patient had another episode of rhabdomyolysis, this time with cardiac arrhythmia. Since the results of NGS panel sequencing and standard cytogenetic analysis were unremarkable, trio exome sequencing was performed. A heterozygous *de novo* missense variant in *BICD2* was detected: *BICD2* (NM_001003800.1): c.2383C>T; p.(Arg795Trp). The disorder *BICD2*-associated spinal muscular atrophy, lower extremity-predominant, 2A (SMALED2A, OMIM

#615290) is characterized by motor developmental delay, muscle weakness, muscle atrophy, and distal hyporeflexia, and has an onset in early infancy. A homozygous deletion in *TANGO2* (NM_152906.5):c.57-1743_*10769del, which is associated with AR recurrent metabolic crises with rhabdomyolysis, cardiac arrhythmias, and neurodegeneration (MECRCN, OMIM #616878), was also identified.

TRDN and *GYG1* (dual diagnosis)

An 8-year-old boy (case 312) with second-degree consanguineous parents was diagnosed with motor developmental delay, suspected cardiomyopathy, and congenital myopathy after resuscitation for ventricular fibrillation was required at the age of 4 years and 3 months. Following Cesarean section in the second stage of labor at 39+2 weeks gestation, the postnatal period was unremarkable. Motor milestones were delayed. He started to turn at the age of 10 months, sit freely and crawl at the age of 16 months, and walk freely at 26 months. Muscular hypotonia was first documented at the age of 6 months. Cognitive and speech development were unremarkable. Since the results of NGS panel sequencing and standard cytogenetic analysis were negative trio exome sequencing was performed. The following homozygous variants were detected: *TRDN* (NM_006073.3):c.508G>T;p.Gly170* and *GYG1* (NM_001184720.1):c.487del; p.Asp163Thrfs*5. Both variants had been described previously [17] and were classified as pathogenic. Biallelic pathogenic variants in *TRDN* are associated with AR cardiac arrhythmia syndrome with or without skeletal muscle weakness (OMIM #615441). Biallelic alterations in *GYG1* are associated with two phenotypically overlapping syndromes: glycogen storage disease 15 (GSDXV, OMIM #613507); and polyglucosan body myopathy type 2 (OMIM #616199). To date, reports in the literature indicate a high degree of clinical variability and no specific genotype-phenotype correlation.

KMT2E and *DDX3X* (locus heterogeneity in two siblings with NDD)

Two siblings (a 15-year-old girl and a 7-year-old boy) whose parents were first cousins presented with a similar phenotype, comprising global developmental delay, muscular hypotonia, and microcephaly. Duo exome sequencing was performed, and the generated data were analyzed under the assumption of an AR disorder, given the parental consanguinity and phenotypic overlap. Duo exome sequencing did not lead to the prioritization of any variants that were shared by both siblings. Therefore, exome sequencing of the parents with combined data analysis was initiated. While a heterozygous *de novo* stop-loss variant in *KMT2E* (NM_018682.3:c.4743_4744del, p.Phe1582Tyrfs*286) was identified in the brother, a heterozygous *de novo* nonsense variant in *DDX3X* (NM_001356.4:c.841C>T, p.Gln281*) was found in the sister, leading to the assignment of two different diagnoses ("O'Donnell-Luria-Rodan syndrome" and “Intellectual developmental disorder, X-linked, syndrome, Snijders Blok type”, respectively). Reverse phenotyping revealed distinct phenotypes in the two children, each of which aligned with the respective diagnosis. This case description is an excellent example of how parental consanguinity poses a pitfall in variant prioritization, particularly in families with more than one affected child. Moreover, it highlights the need for accurate pre-exome phenotyping, as well as the advantage of a trio approach for the detection of *de novo* variants.

KMT2D (functional assays: methylation analysis)

De novo heterozygous missense variants were identified in two individuals, and these were initially classified as “likely pathogenic” on the basis of ACMG guidelines[18]: The first case was a 3-month-old, otherwise healthy girl with neonatal hyperinsulinism. Since congenital hypoglycemia secondary to hyperinsulinism can be a feature of Kabuki Syndrome 1, in-depth phenotyping to detect disease-associated features was recommended by the MDT[19]. However, no Kabuki-like features were evident, thus calling into question a disease

association for the identified variant. The second case was a 2-year-old boy with mild motor delay and muscular hypotonia who was included in TRANSLATE-NAMSE due to the suspicion of a neuromuscular disorder. Similarly, no Kabuki Syndrome-associated features were observed.

A functional assessment of the variants was then performed. In both cases, DNA methylation was analyzed using EPIC arrays, as described elsewhere[19]. This was also performed for n= 5 positive (i.e., confirmed diagnosis of Kabuki syndrome) and n= 97 controls (having other diagnoses or VUS in other genes than *KMT2D*). First, CpG sites that showed a significant epigenome-wide association with Kabuki syndrome were identified via an epigenome-wide association analysis. Second, a support vector machine (SVM) was employed for the epi-signature to distinguish between variants with a Kabuki-like methylation pattern and those without. Interestingly, both cases yielded an SVM score of <0,3 indicating that in general, the variants have no effect on DNA methylation. Based on these findings, the variants were reclassified as VUS in accordance with the ACMG criterion “BS3: Well-established *in vitro* or *in vivo* functional studies show no damaging effect on protein function or splicing”. Of note, Supplementary Figure 10 displays an independent case with an SVM score >0.3 but < 0.5. This case was found to have a pathogenic *KMT2D* variant with high-grade mosaicism in blood DNA.

MDH2 (functional assays: proteomics)

In a 4-year-old girl (case 1127) from a non-consanguineous family who presented with global developmental delay and muscular hypotonia, compound heterozygous VUS c.755C>T, p.Ala252Val and c.884G>T, p.Gly295Val were identified in *MDH2* (NM_005918.2). Additional clinical findings comprised ataxia, stereotypic movements, and growth delay. Serial magnetic resonance imaging studies revealed progressive cerebellar atrophy and delayed myelination. An electroencephalogram revealed frontocentral spikes, although no history of seizures was reported.

The variants in *MDH2* were classified as VUS. Subsequent RNA sequencing from fibroblast RNA revealed no reduction of *MDH2* transcripts and no aberrant splicing. However, subsequent proteomics showed a significant (p=2.45E-09) protein reduction of 60% (log2fold change = -1.316, Supplementary Figure 11A). The protein levels found in the present case were lower than those found in 98 fibroblast proteome samples that were processed in parallel (Supplementary Figure 11B). To date, only three unrelated patients with MDH2 deficiency have been reported in the literature, all of whom presented with a more severe phenotype than the present case[20]. A discussion of molecular and clinical findings within the respective MDT concluded that the variants in *MDH2* were the most likely cause for the encephalopathy in this case, albeit with an attenuated phenotype in comparison to published cases.

RNASEH2A (therapeutic consequences in Aicardi-Goutieres syndrome 4)

A 10-month-old girl (case 892) presented with developmental regression, muscular hypotonia, and an inability to maintain visual fixation. Trio exome sequencing revealed a homozygous missense variant in *RNASEH2A* (NM_006397.2:c.859T>C, p.Tyr287His), which was classified as a VUS in accordance with current ACMG criteria. Subsequent reverse phenotyping generated laboratory findings highly suggestive of “Aicardi-Goutieres syndrome 4” (positive interferon signature in peripheral blood, increased interferon-alpha activity in the cerebrospinal fluid (CSF), CSF leukocytosis) leading to the reclassification of the variant as “likely pathogenic”. Treatment with the JAK inhibitor Ruxolitinib, which is a proposed treatment option for selected interferonopathies, was initiated. This case

illustrates the value of specific laboratory constellations for variant (re-)classification in selected disorders, and the therapeutic consequences of a genetic diagnosis.

GestaltMatcher integration with other tools

The dysmorphic similarity scores from portrait analysis with GestaltMatcher AI can be combined with the results from other prioritization tools. In Figure 5b of the main manuscript the performance of a support vector machine (SVM) is shown that was trained and tested on scores from GestaltMatcher, CADA and CADD. Similar improvements can also be achieved for SVMs that receive scores from Exomiser[21], Xrare[22], LIRICAL[23], and Amelie[24] as input. The addition of GestaltMatcher scores increased the top-1 accuracy by 4 to 38 percentage points and the top-10 accuracy by 6 to 17 percentage points (Supplementary Figure 8).

Supplementary Figures

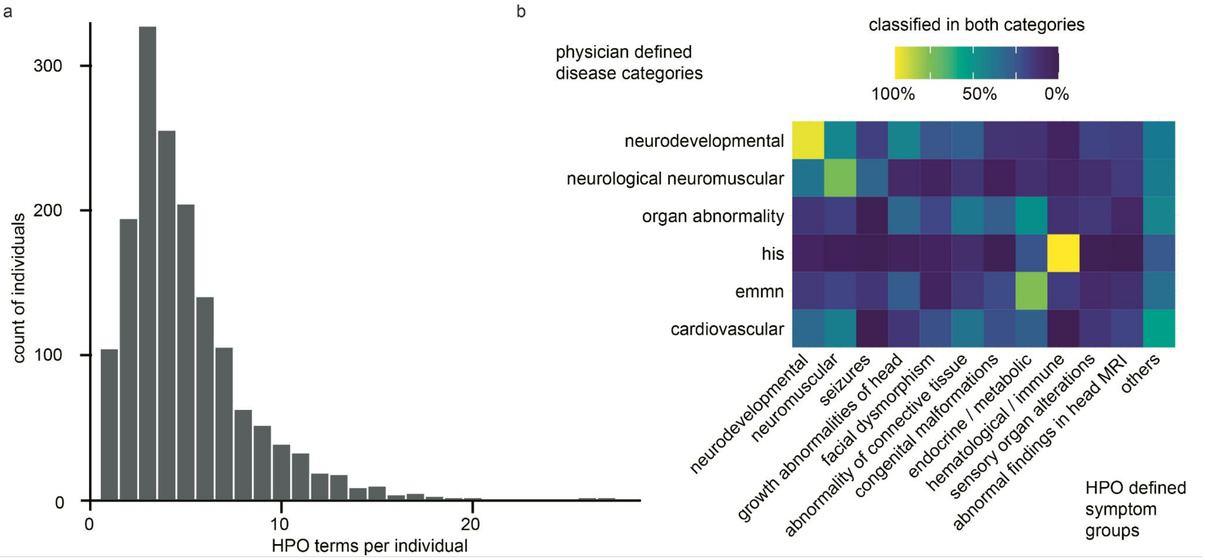

Supplementary Figure 1: HPO terms and correlation with disease groups. a) The mean number of Human Phenotype Ontology (HPO)-terms used to describe a patient was 5. b) Each HPO term was also assigned to a higher-order HPO group in order to study the correlation between the higher-order HPO group and the physician-reported disease groups. A high proportion of individuals with neurodevelopmental disorders also had neurological and neuromuscular abnormalities and vice versa.

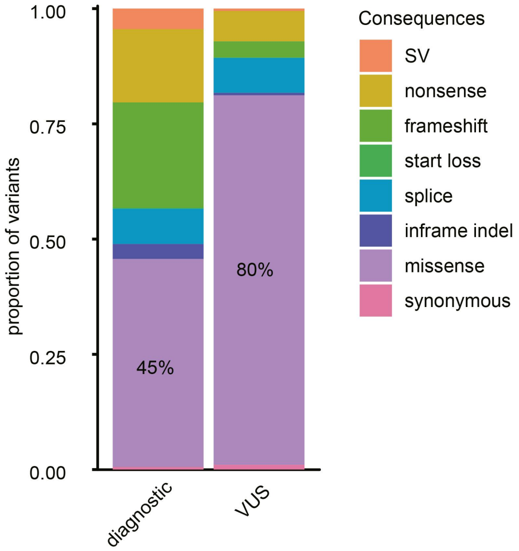

Supplementary Figure 2: Types of diagnostic variants versus types of variants of unknown significance (VUS). VUS were enriched for missense variants (80% vs. 45%, p<0.001). SV: Structural variant; indel: insertion or deletion.

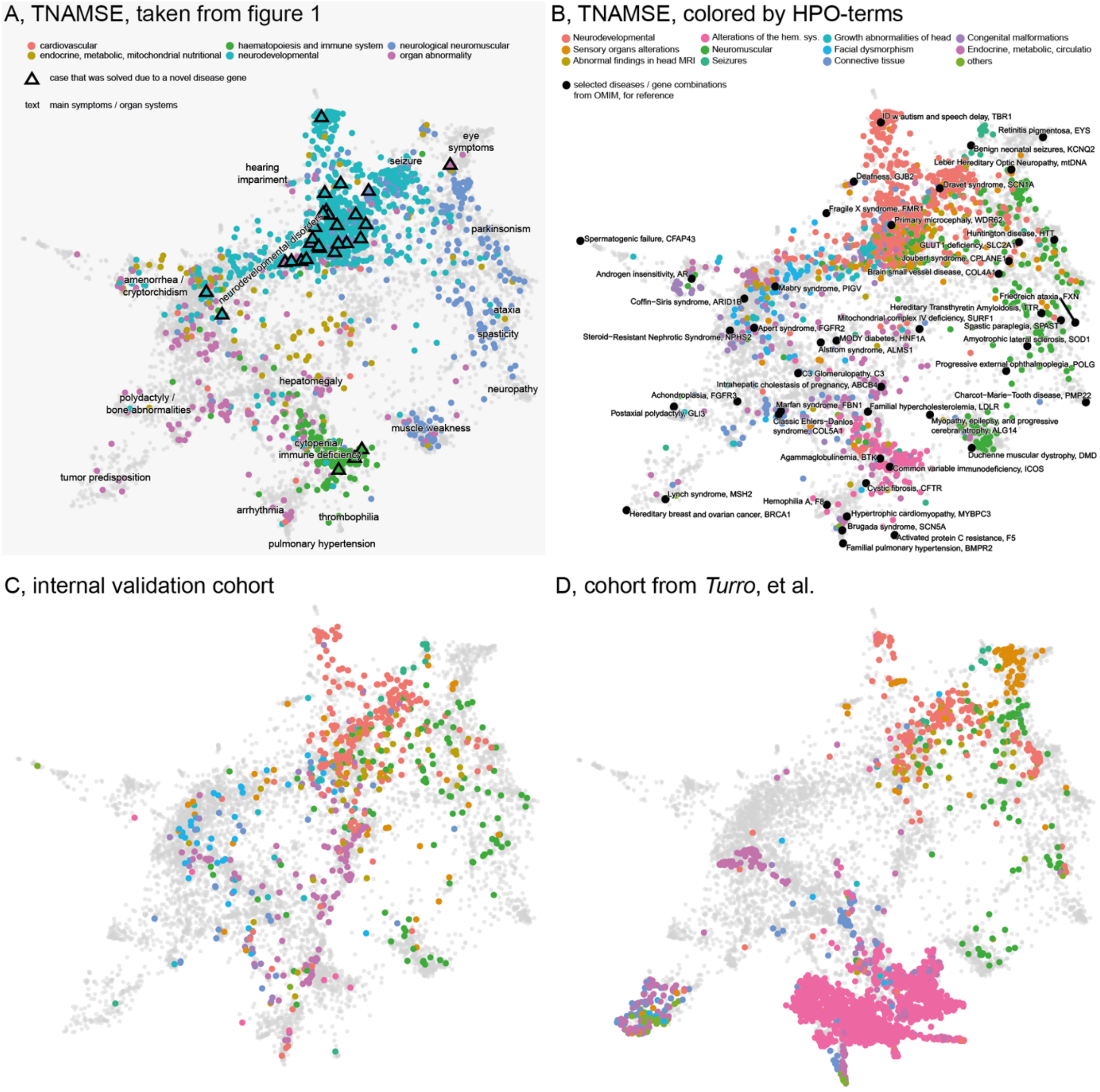

Supplementary Figure 3: Phenotypic similarities between patients of three independent cohorts, as encoded according to their HPO terms, were visualized in analogy to Figure 1c. Panel A is taken from Figure 1c for reference and is color coded by disease category, which is in contrast to panels B-D, which are color coded on the basis of HPO based symptom groups as only HPO terms but no disease categories were available for the cohort from Turro et al. (and as used in Figure 5a for the Lasso-Analysis). The following cohorts are shown: A and B TRANSLATE-NAMSE cohort, C: internal validation cohort, and D: cohort form Turro et al. As additional reference selected monogenic disorders from OMIM and the associated genes were highlighted as black dots for reference in panel B. Note that these dots do not represent patients present in the TRANSLATE-NAMSE cohort. This representation makes some differences between the cohorts evident: The phenotypes described in TRANSLATE-NAMSE and in the internal validation cohort seem to be similar, with a relative enrichment for patients with e.g. abnormalities of the haematopoesis and the immune system in the TRANSLATE-NAMSE cohort. The cohort from Turro, et al. (D) contains several more circumscribed patient groups, such as the large cluster on the bottom, which probably contains the three largest domains of phenotypes described in the corresponding publication (bleeding, thrombotic and platelet disorders; pulmonary arterial hypertension, and primary immune disorders). Additionally, in particular tumor and eye diseases were more prevalent in Turro et al., in comparison to the TRANSLATE-NAMSE cohort.

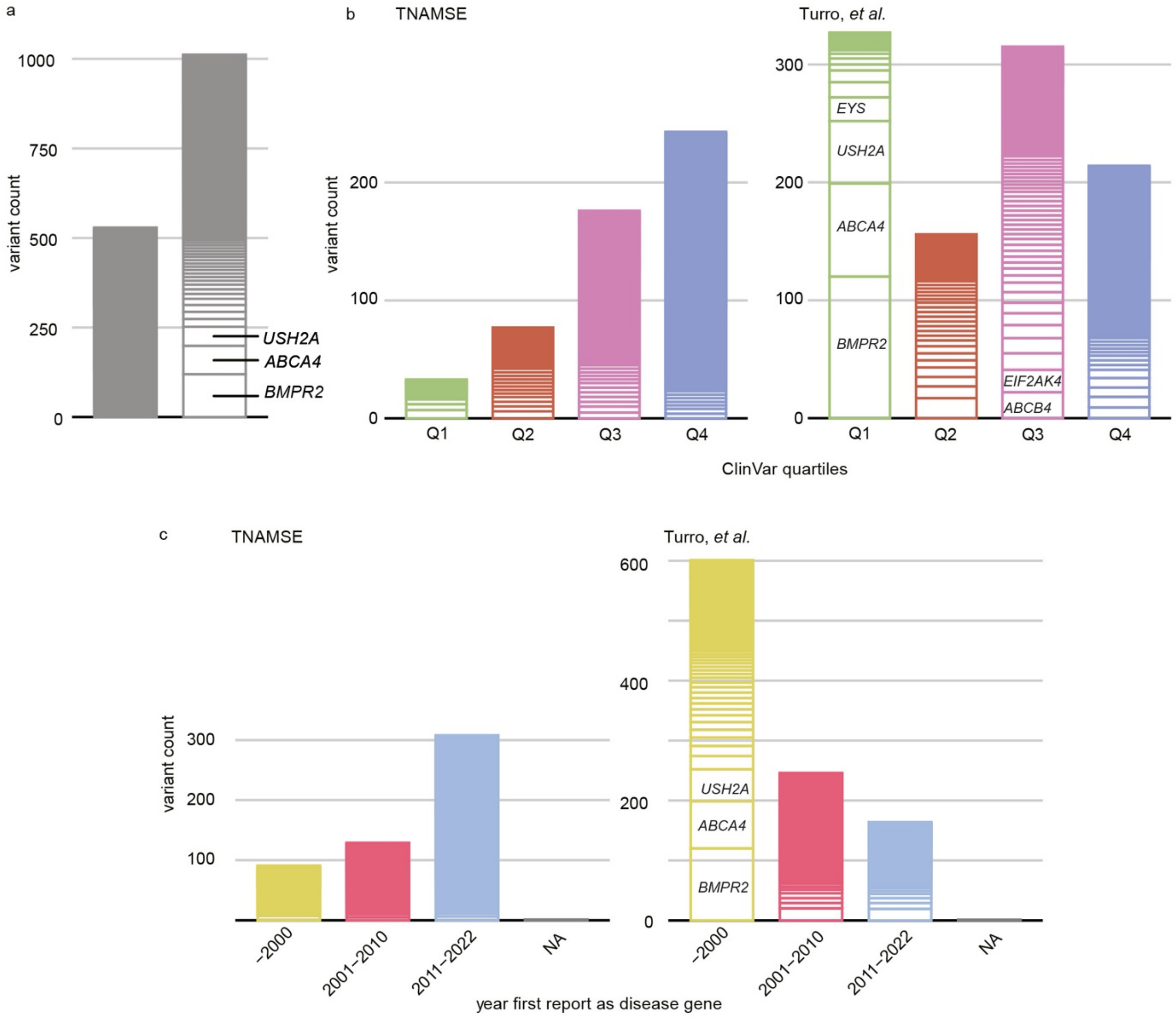

Supplementary Figure 4: Comparison of molecular diagnoses in the rare disease programs of two different national health systems (TRANSLATE NAMSE, Germany; and NIHR BioResource from Turro, et al., UK[25]). A) The total number of (likely) pathogenic variants reported by Turro, et al. was larger. However, many of these were in DGGs that are subject in Germany to tests such as panel testing. B) Disease-associated genes were ranked according to the number of (likely) pathogenic variants submitted to ClinVar, with the most frequently affected genes in the 1st quartile and the least frequently affected genes in the 4th. The majority of (likely) pathogenic variants reported in TRANSLATE NAMSE were in disease-associated genes from the 4th quartile. C) The causative disease-associated genes found in TRANSLATE NAMSE and by Turro, et al. were sorted according to the year in which they were first reported as disease-associated genes. The different distributions illustrate the high proportion of recently described disease-associated genes in the TRANSLATE NAMSE exome sequencing cohort. NA corresponds to genes that did not reach disease-associated gene status prior to the publication of the present study. Note that 59 genes listed in the recommendations for reporting of secondary findings (version 2) of the American College of Medical Genetics were excluded from the analyses to counteract potential biases in ClinVar due to submissions of secondary findings. However, sensitivity analyses indicated that the observation of a high proportion of variants in Q4 within the TRANSLATE NAMSE cohort is not sensitive to inclusion or exclusion of these 59 genes.

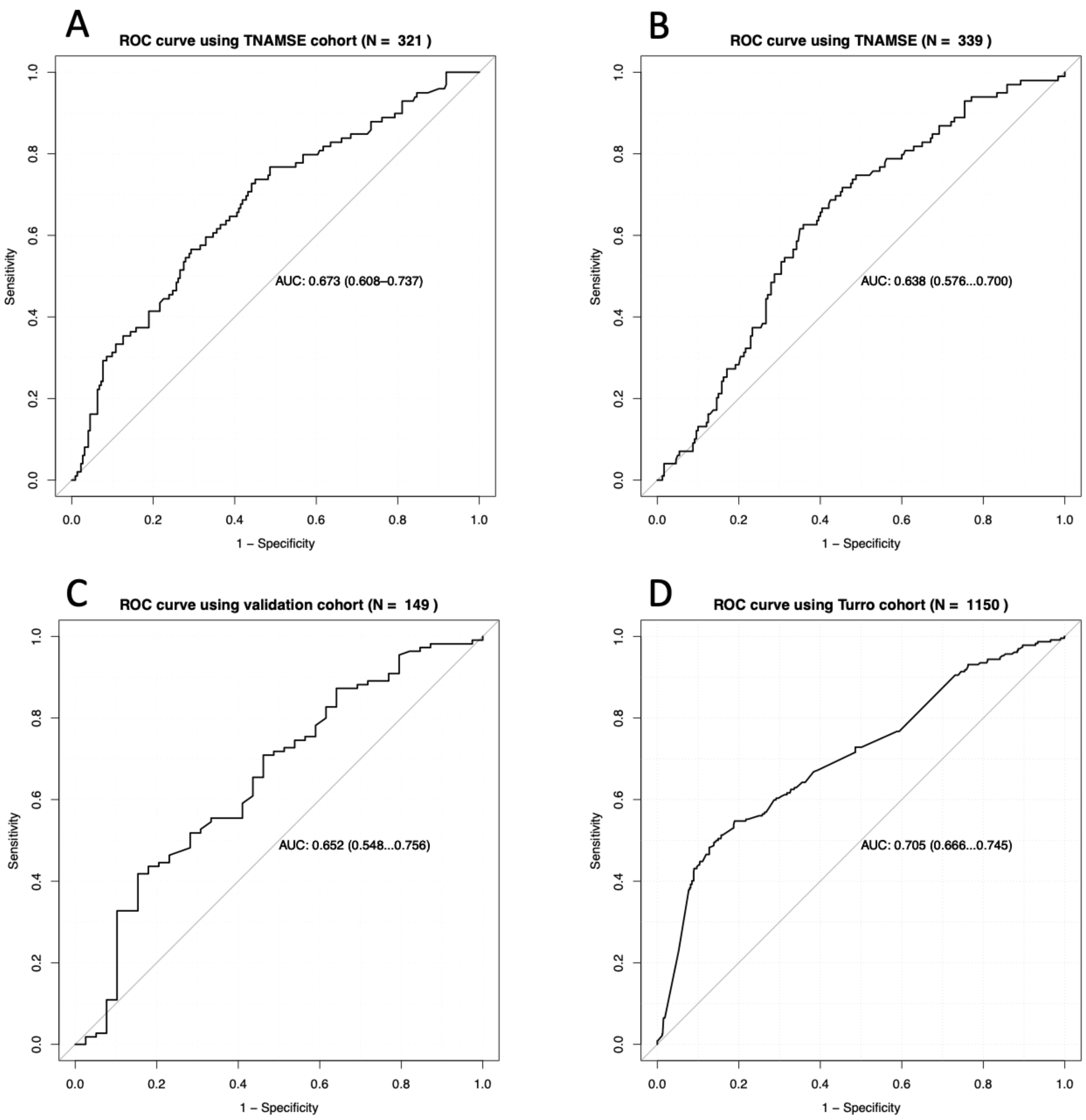

Supplementary Figure 5: Receiver operator characteristic (ROC) curves for the Lasso model that was only trained and tested on cases of the TRANSLATE NAMSE cohort (A) and second Lasso model that was trained and tested on cases of three cohorts (B-D). For testing, 20% hold-out sets were used. For the new model the AUC for the TRANSLATE NAMSE test set drops slightly from 0.67 to 0.64. However, the generalization is markedly improved, achieving an AUC of 0.65 on the test set of the validation cohort and 0.71 on the test set of the NIHR BioResource cohort described by Turro, et al.

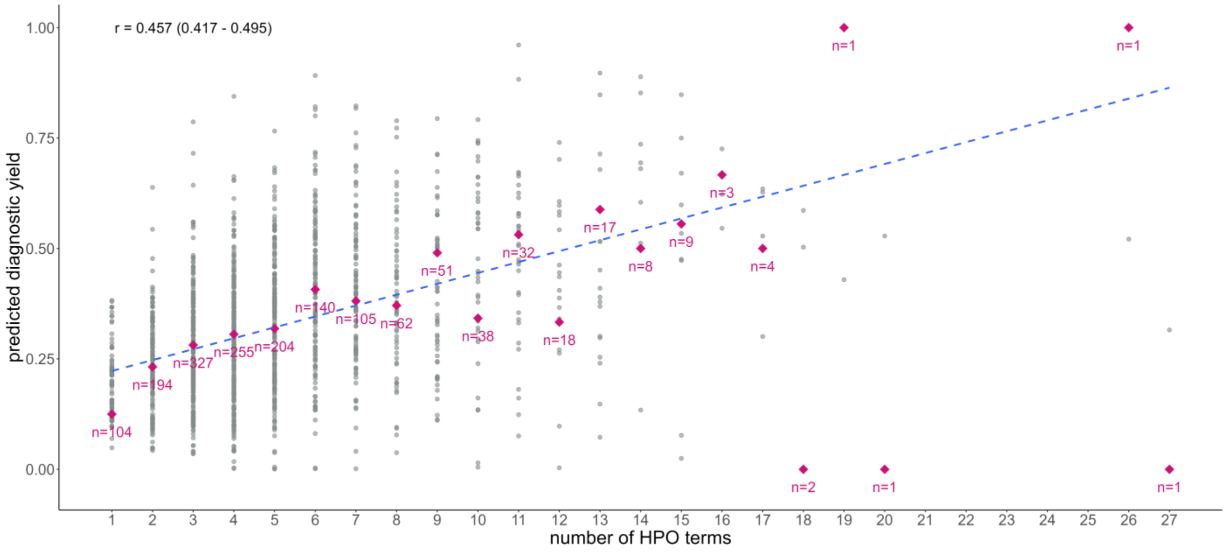

Supplementary Figure 6: The count of HPO-terms that was used to describe a case is correlated with the diagnostic yield. The predicted (grey dots) as well as the average observed diagnostic yield (red square) is given in relation to the number of HPO-terms of cases in TRANSLATE NAMSE. A linear regression was fitted to the predicted diagnostic yields (blue dashed line). The number of cases with a certain number of HPO-terms is given as red text.

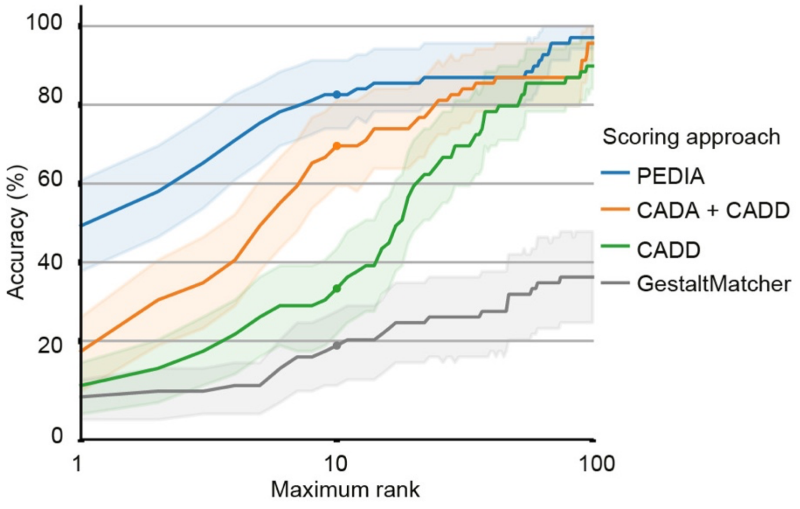

Supplementary Figure 7: Performance of selected variant prioritization approaches. The validation cohort was used to compare the performance of selected variant prioritization approaches. All disease-associated genes were ranked using the respective variant prioritization method. Subsequently, the proportion of cases detected with the correct disease-associated gene (sensitivity) was shown as a function of the number of disease-associated genes considered, beginning at the top score. The following three approaches for variant prioritization were then tested in solved cases from the PEDIA validation cohort (n=69): 1) only a molecular pathogenicity score (CADD) with a top-10 accuracy of 33%; 2) a feature-based score (CADA) in addition to CADD with a top-10 accuracy of 68%; 3) A gestalt score from facial image analysis (GestaltMatcher) in addition to both CADD and CADA – termed the PEDIA score - with a top-10 accuracy of 83% Note that the bold lines indicate the observed top-k accuracy and bootstrapped 95% confidence intervals are indicated by the lighter shading around the lines. CADA: Case Annotation and Disorder Annotation; CADD: Combined Annotation-Dependent Depletion; PEDIA: Prioritization of Exome Data by Image Analysis

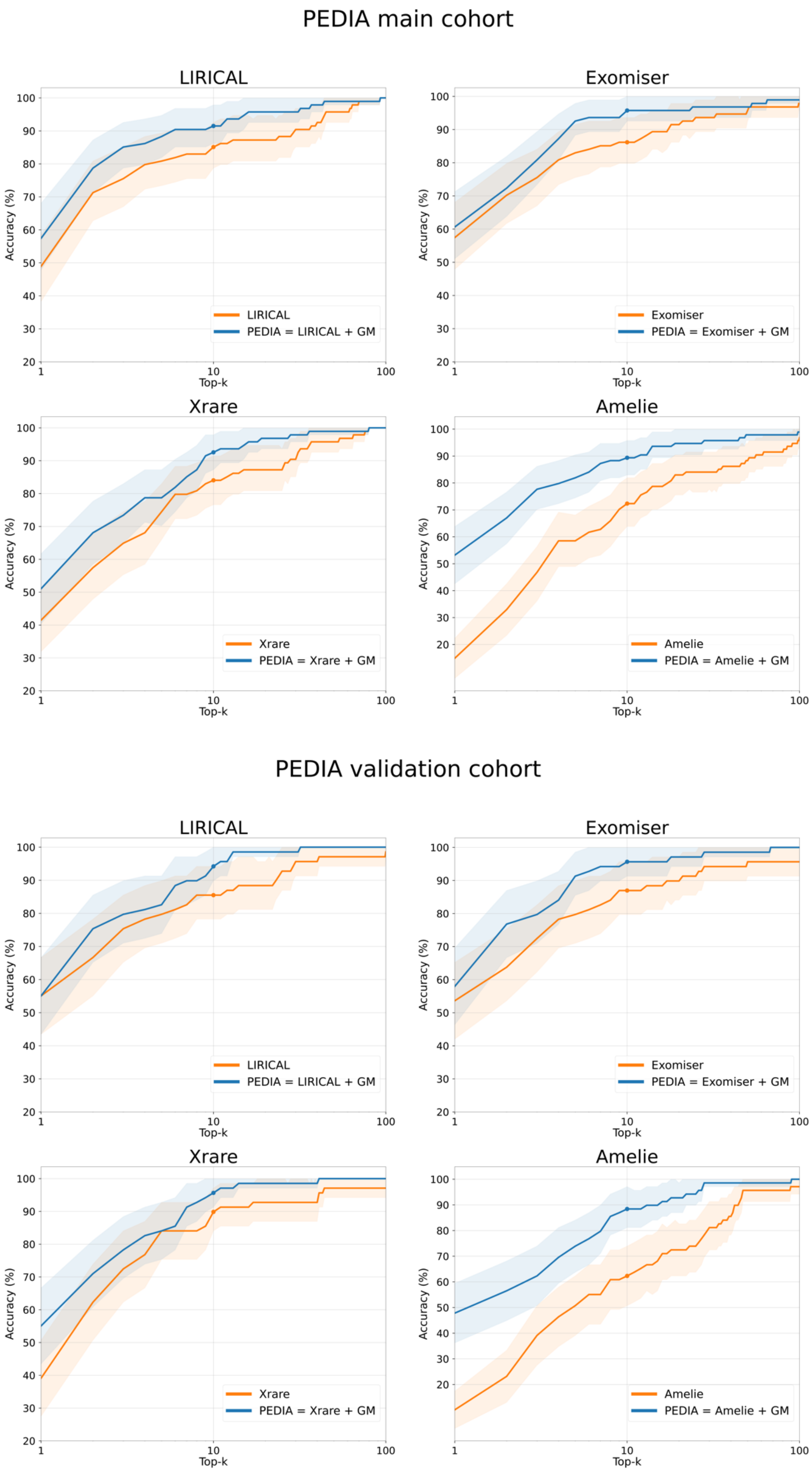

Supplementary Figure 8: Gestalt scores improve the accuracy of prioritization tools. Bold lines indicate the observed top-k accuracy and bootstrapped 95% confidence intervals are indicated by the lighter shading around the lines. GM: GestaltMatcher.

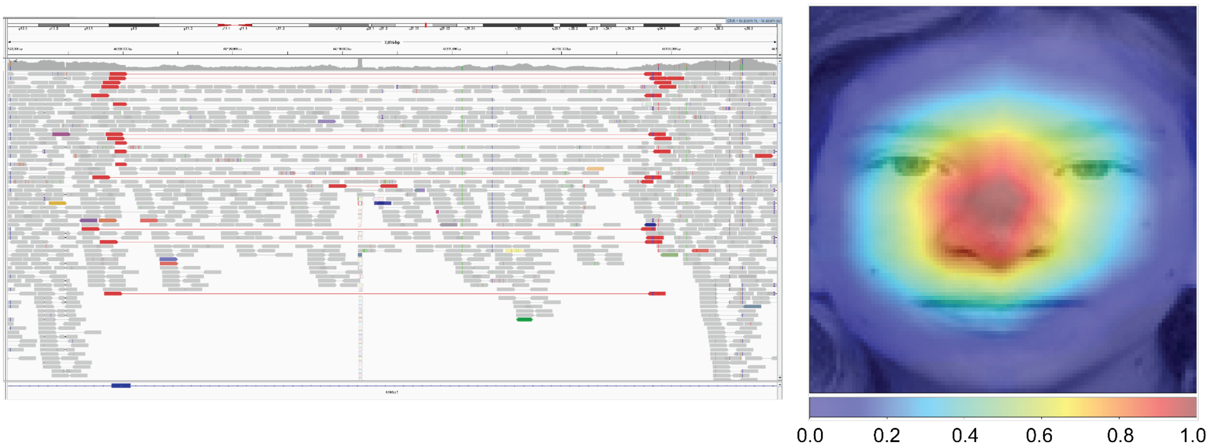

Supplementary Figure 9: Value of next-generation phenotyping. Facial image analysis of case 393 with GestaltMatcher suggested a high similarity to Koolen-De-Vries syndrome. After inconclusive exome sequencing, analyses targeted at KANSL1 and genome sequencing were performed. These revealed a causal 4.7 kb de novo deletion in KANSL1[16].

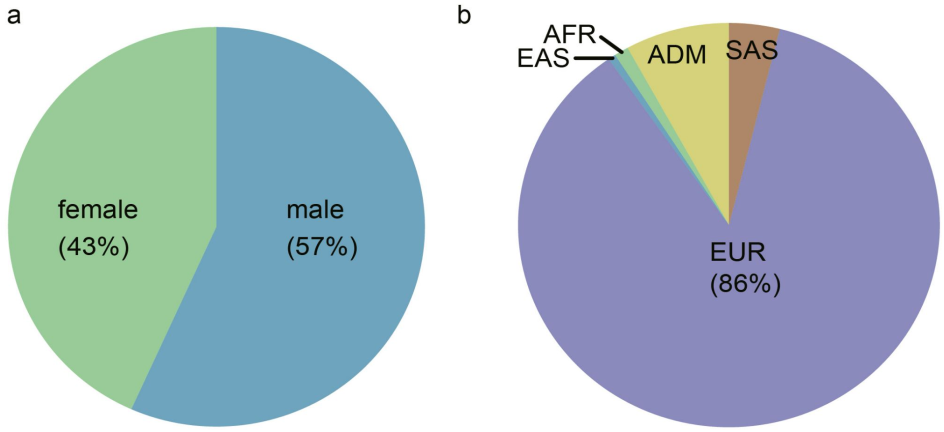

Supplementary Figure 10: Sex and population background of the TRANSLATE NAMSE exome sequencing cohort. Pie chart showing (a) the sex of 1,577 individuals from the TRANSLATE NAMSE exome sequencing cohort and (b) the available population background of 1,365 individuals. As expected, the majority of individuals (86% or 1,180) were assigned to the European population. EAS: East Asian; AFR: African; ADM: admixed; SAS: South Asian; EUR: European.

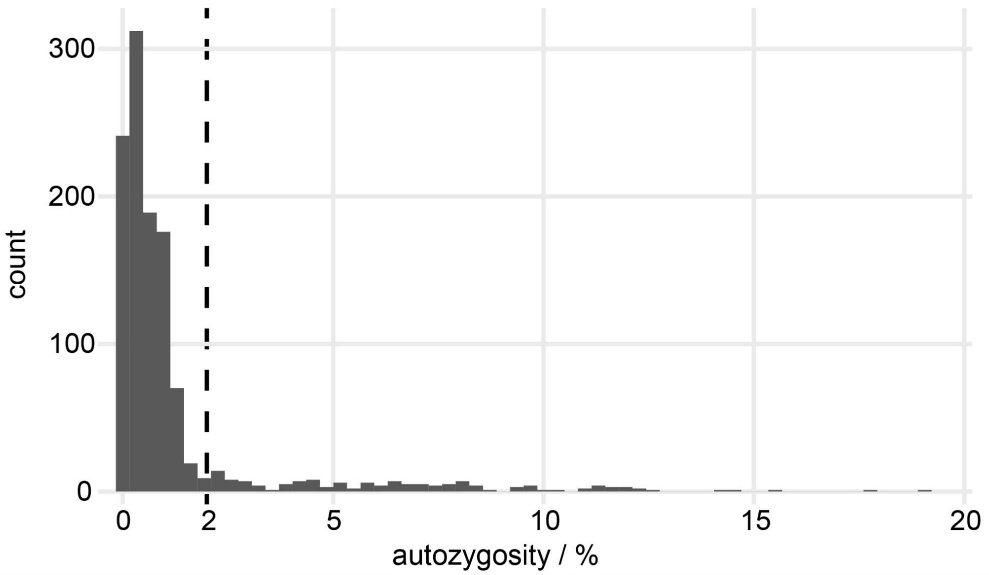

Supplementary Figure 11: ***Histogram showing the autozygosity distribution*** *in the subcohort of n=1,158 individuals for whom autozygosity data were obtained. The x-axis represents the autozygosity, and the y-axis represents the count of individuals with the respective autozygosity. The vertical line indicates the 2% cut-off used to classify individuals as having low or high autozygosity.*

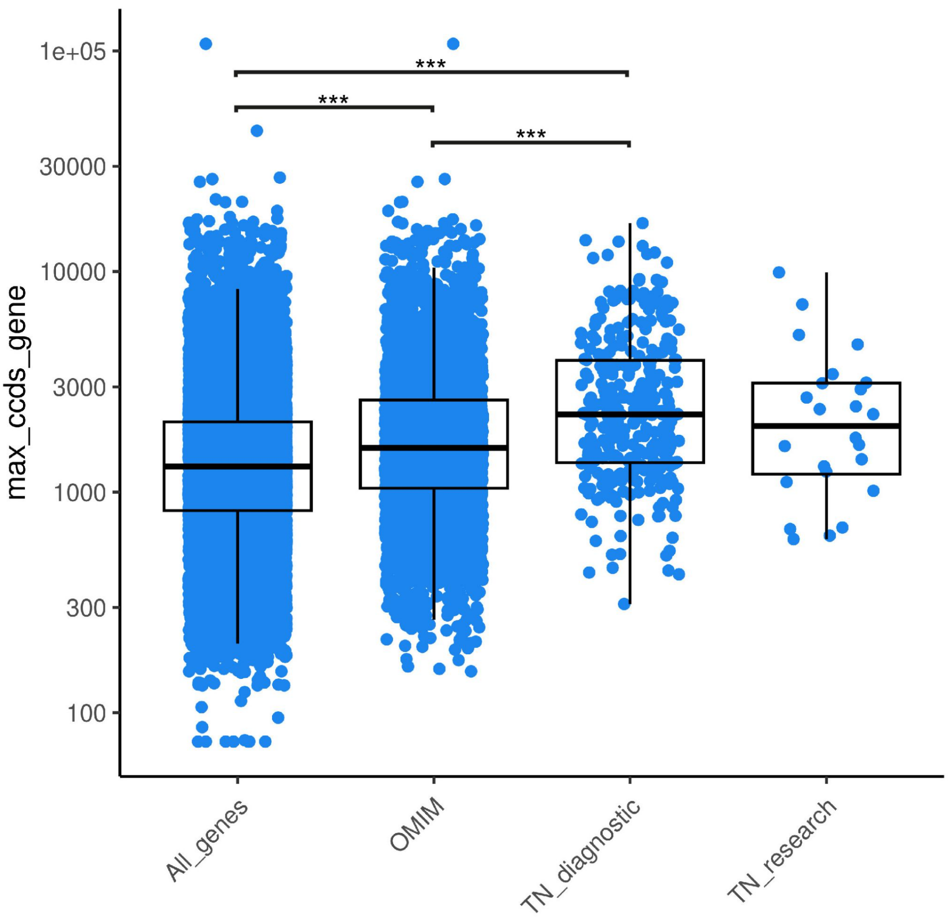

*Supplementary Figure 12: Disease genes in TRANSLATE NAMSE cohort are significantly longer than all genes/OMIM genes. Boxplots of the coding length of different gene sets are shown. The mean coding length of disease-associated genes in the TRANSLATE NAMSE (TN) exome sequencing cohort, which was subdivided into known disease-associated genes (TN_diagnostic, n=330) and novel disease-associated genes (TN-research, n=24), was significantly longer than for all genes (n=18,598) or for all OMIM genes (n=4,369; pairwise two-sided t-test. P-values were adjusted by Bonferroni correction. *** = p<0.001, corrected p-values: all genes vs. OMIM p<2*10^-16, all genes vs. TN_diagnostic p<2*10^-16, OMIM vs. TN_diagnostic p=3.3*10^-13). In the box plots, the center lines indicate the median values, and the bottom and top edges of the boxes are the first (25%) and the third (75%) quartiles. The whiskers extend to the minimal and maximal data points with a maximum distance of 1.5 interquartile ranges from the edges of the box.*

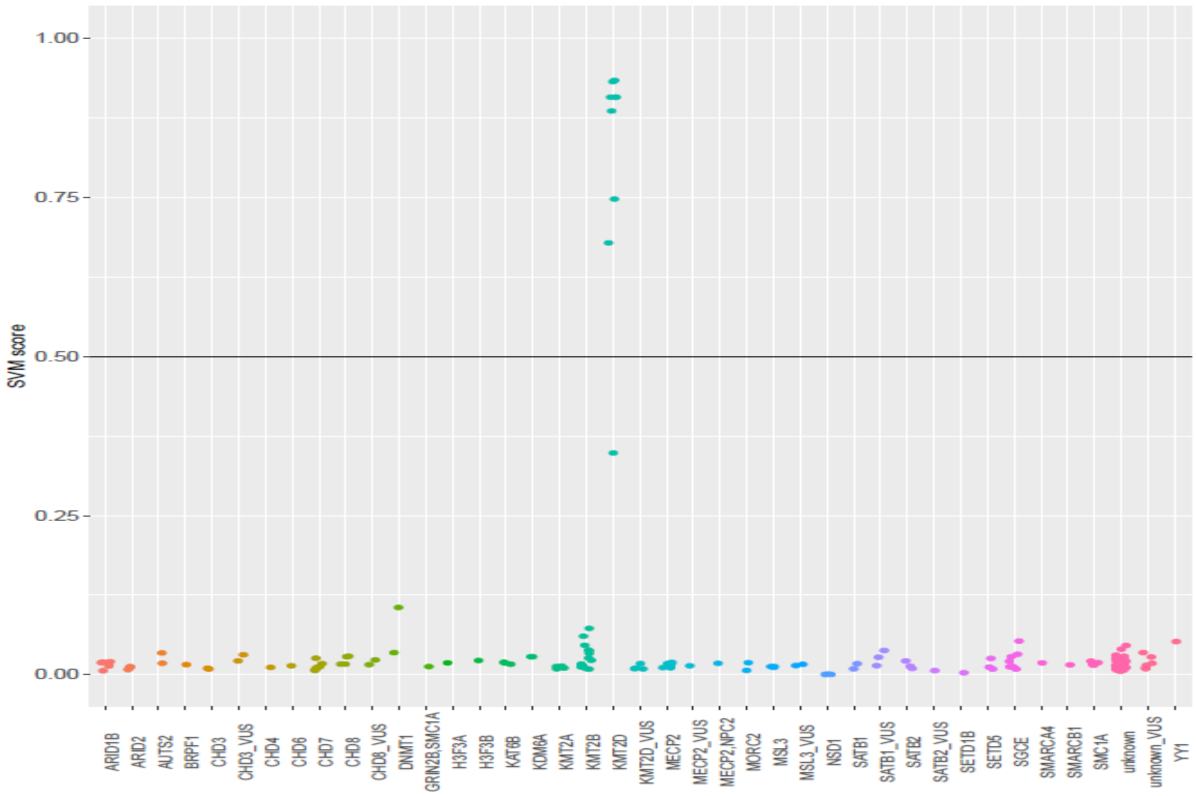

Supplementary Figure 13: Monogenic disorders of chromatin regulatory proteins cause characteristic methylation epi-signatures. These patterns could be identified using EPIC-array data and a support vector machine (SVM) that was trained on positive controls for each gene. A score above 0.5 is indicative of a pathogenic variant in the respective gene. Scores of between 0.3 and 0.5 can be caused by mosaicism of pathogenic variants. Individuals with de novo variants in KMT2D (see Case reports of particular interest), formerly classified as "likely pathogenic" had a SVM score < 0.3 (here displayed as KMT2D_VUS) excluding an effect of these variants on methylation. Thus, variants could be reclassified as VUS.

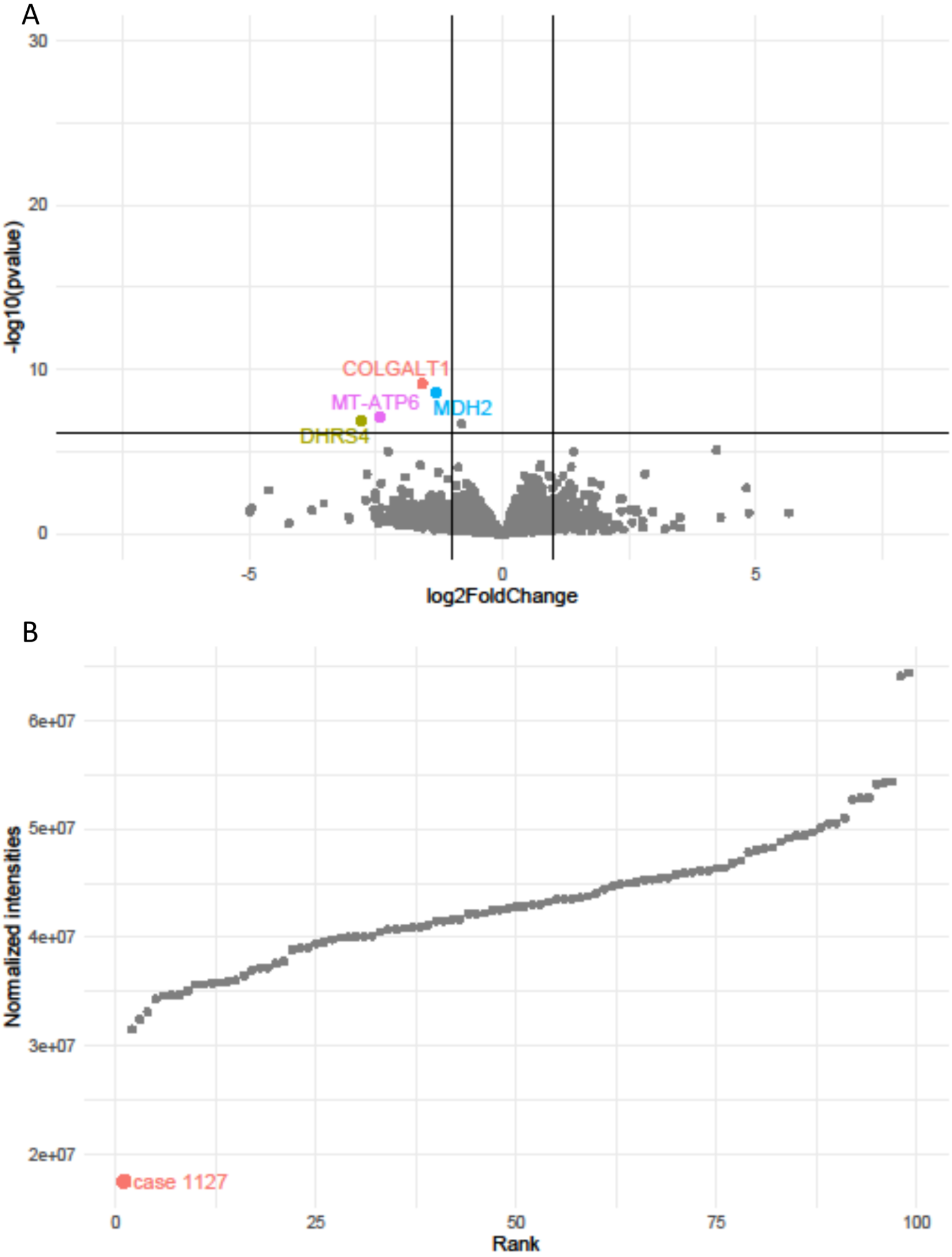

*Supplementary Figure 14: Significant MDH2 reduction in fibroblasts. Exome sequencing in case 1127 revealed compound-heterozygous missense variants of uncertain clinical significance in MDH2. Subsequent proteomics in fibroblasts showed significantly reduced intensities for MDH2 (A) with a log2fold change of -1.316 (p= 2.45E-09, two-sided testing). P values were calculated as previously described[26]. Vertical black lines indicate log2fold changes of -1 and 1. Horizontal black lines depict significance level of p = 7,14*10-6 (Bonferroni correction for 7000 hypotheses representing the number of proteins identified). Proteins with statistically different levels are highlighted in colour. This resulted in a reclassification of the variants to likely pathogenic. Off note, proteomics analysis detected an outlier, LAGE3 (log2fold change -7.116, p= 9.73E-46) which however is a false positive as a rare SNP affects a peptide which is subsequently undetectable by mass spectroscopy. (B) MDH2 had the lowest normalized intensity in proteomics in comparison with 98 controls.*

Supplementary Tables

Supplementary Table 1: Complete TRANSLATE NAMSE exome sequencing dataset, comprising information on all 1,577 cases included in the present study. Due to its size, the table has been submitted separately.

Supplementary Table 2: Dual diagnoses

Supplementary Table 3: Candidate genes and novel disease-associated genes

Supplementary Table 4: Description of the analysis pipelines of the five laboratories

Supplementary Table 5: YieldPred validation cohort

Supplementary Table 6: PEDIA validation cohort

Supplementary Table 7: Secondary findings

References

1. Li, D. *et al.* Pathogenic variants in *SMARCA5*, a chromatin remodeler, cause a range of syndromic neurodevelopmental features. *Sci Adv* **7**, (2021).
2. Mochel, F. *et al.* Variants in the SK2 channel gene (KCNN2) lead to dominant neurodevelopmental movement disorders. *Brain* **143**, 3564–3573 (2020).
3. Horn, D. *et al.* Biallelic truncating variants in MAPKAPK5 cause a new developmental disorder involving neurological, cardiac, and facial anomalies combined with synpolydactyly. *Genet. Med.* **23**, 679–688 (2021).
4. Gambin, T. *et al.* Low-level parental somatic mosaic SNVs in exomes from a large cohort of trios with diverse suspected Mendelian conditions. *Genet. Med.* **22**, 1768–1776 (2020).
5. Wright, C. F. *et al.* Clinically-relevant postzygotic mosaicism in parents and children with developmental disorders in trio exome sequencing data. *Nat. Commun.* **10**, 2985 (2019).
6. Cao, Y. *et al.* A clinical survey of mosaic single nucleotide variants in disease-causing genes detected by exome sequencing. *Genome Med.* **11**, 48 (2019).
7. Green, R. C. *et al.* ACMG recommendations for reporting of incidental findings in clinical exome and genome sequencing. *Genet. Med.* **15**, 565–574 (2013).
8. Kalia, S. S. *et al.* Recommendations for reporting of secondary findings in clinical exome and genome sequencing, 2016 update (ACMG SF v2.0): a policy statement of the American College of Medical Genetics and Genomics. *Genet. Med.* **19**, 249–255 (2017).
9. Daly, M. B. *et al.* NCCN Guidelines Insights: Genetic/Familial High-Risk Assessment: Breast and Ovarian, Version 2.2017. *J. Natl. Compr. Canc. Netw.* **15**, 9–20 (2017).
10. Luzzatto, L., Nannelli, C. & Notaro, R. Glucose-6-Phosphate Dehydrogenase Deficiency. *Hematol. Oncol. Clin. North Am.* **30**, 373–393 (2016).
11. Yang, Y. *et al.* Molecular findings among patients referred for clinical whole-exome sequencing. *JAMA* **312**, 1870–1879 (2014).

12. Dorschner, M. O. *et al.* Actionable, pathogenic incidental findings in 1,000 participants' exomes. *Am. J. Hum. Genet.* **93**, 631–640 (2013).
13. Johnston, J. J. *et al.* Secondary variants in individuals undergoing exome sequencing: screening of 572 individuals identifies high-penetrance mutations in cancer-susceptibility genes. *Am. J. Hum. Genet.* **91**, 97–108 (2012).
14. Cassa, C. A., Tong, M. Y. & Jordan, D. M. Large numbers of genetic variants considered to be pathogenic are common in asymptomatic individuals. *Hum. Mutat.* **34**, 1216–1220 (2013).
15. Ding, L.-E., Burnett, L. & Chesher, D. The impact of reporting incidental findings from exome and whole-genome sequencing: predicted frequencies based on modeling. *Genet. Med.* **17**, 197–204 (2015).
16. Brand, F. *et al.* Next-generation phenotyping contributing to the identification of a 4.7 kb deletion in KANSL1 causing Koolen-de Vries syndrome. *Hum. Mutat.* **43**, 1659–1665 (2022).
17. Desikan, M. *et al.* GYG1 causing progressive limb girdle myopathy with onset during teenage years (polyglucosan body myopathy 2). *Neuromuscul. Disord.* **28**, 346–349 (2018).
18. Richards, S. *et al.* Standards and guidelines for the interpretation of sequence variants: a joint consensus recommendation of the American College of Medical Genetics and Genomics and the Association for Molecular Pathology. *Genet. Med.* **17**, 405–424 (2015).
19. Yap, K. L. *et al.* Congenital hyperinsulinism as the presenting feature of Kabuki syndrome: clinical and molecular characterization of 9 affected individuals. *Genet. Med.* **21**, 233–242 (2019).
20. Ait-El-Mkadem, S. *et al.* Mutations in MDH2, Encoding a Krebs Cycle Enzyme, Cause Early-Onset Severe Encephalopathy. *Am. J. Hum. Genet.* **100**, 151–159 (2017).
21. Robinson, P. N. *et al.* Improved exome prioritization of disease genes through cross-species phenotype comparison. *Genome Res.* **24**, 340–348 (2014).

22. Li, Q., Zhao, K., Bustamante, C. D., Ma, X. & Wong, W. H. Xrare: a machine learning method jointly modeling phenotypes and genetic evidence for rare disease diagnosis. *Genet. Med.* **21**, 2126–2134 (2019).
23. Robinson, P. N. *et al.* Interpretable Clinical Genomics with a Likelihood Ratio Paradigm. *Am. J. Hum. Genet.* **107**, 403–417 (2020).
24. Birgmeier, J. *et al.* AMELIE speeds Mendelian diagnosis by matching patient phenotype and genotype to primary literature. *Sci. Transl. Med.* **12**, (2020).
25. Turro, E. *et al.* Whole-genome sequencing of patients with rare diseases in a national health system. *Nature* **583**, 96–102 (2020).
26. Kopajtich, R. *et al.* Integration of proteomics with genomics and transcriptomics increases the diagnostic rate of Mendelian disorders. *medRxiv* 2021.03.09.21253187 (2021) doi:10.1101/2021.03.09.21253187.
