# Supplementary material for: Next-generation phenotyping integrated in a national framework for patients with ultrarare disorders improves genetic diagnostics and yields new molecular findings
Source: Nat Genet. 2024 Jul 22;56(8):1644–53. doi: 10.1038/s41588-024-01836-1 (PMC11319204; doi:10.1038/s41588-024-01836-1)
Supplement: Supplementary file 2 — Reporting Summary [file 41588_2024_1836_MOESM2_ESM.pdf]

nature portfolio

Corresponding author(s): Peter Krawitz

Last updated by author(s): Mar 27, 2024

Reporting Summary

Nature Portfolio wishes to improve the reproducibility of the work that we publish. This form provides structure for consistency and transparency in reporting. For further information on Nature Portfolio policies, see our Editorial Policies and the Editorial Policy Checklist.

Statistics

For all statistical analyses, confirm that the following items are present in the figure legend, table legend, main text, or Methods section.

n/a	Confirmed	
☐	☒	The exact sample size (*n*) for each experimental group/condition, given as a discrete number and unit of measurement
☐	☒	A statement on whether measurements were taken from distinct samples or whether the same sample was measured repeatedly
☐	☒	The statistical test(s) used AND whether they are one- or two-sided<br>*Only common tests should be described solely by name; describe more complex techniques in the Methods section.*
☐	☒	A description of all covariates tested
☐	☒	A description of any assumptions or corrections, such as tests of normality and adjustment for multiple comparisons
☐	☒	A full description of the statistical parameters including central tendency (e.g. means) or other basic estimates (e.g. regression coefficient) AND variation (e.g. standard deviation) or associated estimates of uncertainty (e.g. confidence intervals)
☐	☒	For null hypothesis testing, the test statistic (e.g. *F*, *t*, *r*) with confidence intervals, effect sizes, degrees of freedom and *P* value noted<br>*Give P values as exact values whenever suitable.*
☐	☒	For Bayesian analysis, information on the choice of priors and Markov chain Monte Carlo settings
☐	☒	For hierarchical and complex designs, identification of the appropriate level for tests and full reporting of outcomes
☐	☒	Estimates of effect sizes (e.g. Cohen's *d*, Pearson's *r*), indicating how they were calculated

Our web collection on statistics for biologists contains articles on many of the points above.

Software and code

Policy information about availability of computer code

Data collection	For individuals additionally participating in the PEDIA study, scores of the analysis of the portrait images by artificial intelligence (PEDIA v3) were collected via the PEDIA web service (https://www.pedia-study.org/).
Data analysis	All analyses that have been conducted on the data are described in the main manuscript and the supplement. The regarding code can be found in the publicly available github respository cited in the manuscript (https://github.com/Ax-Sch/TNAMSE_geno_pheno). The analyses were conducted in the statistics software R (version 4.2.2).<br>Raw exome data were analysed with BWA v0.7.11 through to BWA-Mem v0.7.17, HaplotypeCaller (v3.7, v3.8 or v4.1), Freebayes (v1.2.0), SAMtools v.0.1.7, ExomeDepth v1.1.10 , ClinCNV v.1.16.1, ngs-bits v2019_09, VEP v96 and Jannovar v0.24.

For manuscripts utilizing custom algorithms or software that are central to the research but not yet described in published literature, software must be made available to editors and reviewers. We strongly encourage code deposition in a community repository (e.g. GitHub). See the Nature Portfolio guidelines for submitting code & software for further information.

Data

Policy information about availability of data

All manuscripts must include a data availability statement. This statement should provide the following information, where applicable:

- Accession codes, unique identifiers, or web links for publicly available datasets
- A description of any restrictions on data availability
- For clinical datasets or third party data, please ensure that the statement adheres to our policy

The corresponding author agrees to fulfill any requests for materials not included in the extended data or Supplementary Material, subject to verification that the request adheres to the consent provided by the research participants. Patient-related data not included in the article may be subject to patient confidentiality. Raw

sequencing data was not consented for sharing, except for the PEDIA subset which is available upon request. Reported alleles and their clinical interpretation have been deposited in ClinVar using the following submitters:

Institute for Genomic Statistics and Bioinformatics (University Hospital Bonn):
https://www.ncbi.nlm.nih.gov/clinvar/submitters/507028/,
https://www.ncbi.nlm.nih.gov/clinvar/submitters/508040/
Institute of Human Genetics, Klinikum rechts der Isar (Technical University Munich):
https://www.ncbi.nlm.nih.gov/clinvar/submitters/500240/,
Institute for Medical Genetics and Human Genetics (Charité- Universitätsmedizin Berlin):
https://www.ncbi.nlm.nih.gov/clinvar/submitters/505735/,
Institute of Medical Genetics and Applied Genomics (University Hospital Tübingen):
https://www.ncbi.nlm.nih.gov/clinvar/submitters/506385/.
Genomics Facility (Ludwig-Maximilians-Universität München):
https://www.ncbi.nlm.nih.gov/clinvar/submitters/507363/

Field-specific reporting

Please select the one below that is the best fit for your research. If you are not sure, read the appropriate sections before making your selection.

☒ Life sciences ☐ Behavioural & social sciences ☐ Ecological, evolutionary & environmental sciences

For a reference copy of the document with all sections, see nature.com/documents/nr-reporting-summary-flat.pdf

Life sciences study design

All studies must disclose on these points even when the disclosure is negative.

Sample size	In total, 5652 individuals with a suspected rare disorder were enrolled in TRANSLATE-NAMSE by centers for rare diseases at ten German university hospitals over a period of three years (2018-2020), i.e. over the complete duration of the study. A cohort of 1577 patients underwent exome sequencing with recommendation of multidisciplinary teams. In this manuscript we report results for those 1577 patients of which 211 patients additionally consented to the analysis of their potraits by an artificial intelligence tool (PEDIA). For the evaluation of the exome sequencing cohort, no sample size calculation was performed beforehand, but all eligible samples were included in the study and there was no option to further increase the sample size. The achieved sample size was considered sufficient for descriptive statistics. For the final predictive model (YieldPred), additional cohorts were included to increase the sample size (NIHR BioResource, n=5,510 and external validation cohort, n=753).
Data exclusions	No data were excluded.
Replication	All findings of the analysis can be reproduced by the provided code (https://github.com/Ax-Sch/TNAMSE_geno_pheno). We validated the findings of the LASSO model on an external patient cohort (shown in the supplement) and the NIHR BioResource.
Randomization	For the LASSO analysis the patients were randomly assigned to training (80%) and test (20%) data sets. All other analyses were conducted on the complete TRANSLATE-NAMSE exome sequencing cohort.
Blinding	Splitting data into training and test sets is equivalent to blinding data for the machine.

Reporting for specific materials, systems and methods

We require information from authors about some types of materials, experimental systems and methods used in many studies. Here, indicate whether each material, system or method listed is relevant to your study. If you are not sure if a list item applies to your research, read the appropriate section before selecting a response.

Materials & experimental systems

n/a	Involved in the study	
☒	☐	Antibodies
☒	☐	Eukaryotic cell lines
☒	☐	Palaeontology and archaeology
☒	☐	Animals and other organisms
☐	☒	Human research participants
☒	☐	Clinical data
☒	☐	Dual use research of concern

Methods

n/a	Involved in the study	
☒	☐	ChIP-seq
☒	☐	Flow cytometry
☒	☐	MRI-based neuroimaging

Human research participants

Policy information about studies involving human research participants

Population characteristics	Male and female as well as pediatric and adult participants were analyzed in the study. All patients underwent exome sequencing while a subset of patients additionally consented to image analysis of their potraits by artificial intelligence (PEDIA). Age (below or above 18 years), sex, the sequencing laboratory as well as the use of the PEDIA workflow could confound the diagnostic yield and the data were therefore included as confounders in the LASSO analysis.
Recruitment	Participants were enrolled in the TRANSLATE-NAMSE study at ten centers for rare diseases at German university hospitals and recommended to undergo exome sequencing by multidisciplinary teams.
Ethics oversight	This study is governed by the approval of the following Institutional Review Boards: Charité–Universitätsmedizin Berlin, Germany (EA2/140/17); UKB Universitätsklinikum Bonn, Germany (Lfd.Nr.386/17); Universitätsklinikum Essen, University Duisburg-Essen, Germany (17-7774-BO); Universitätsklinikum Heidelberg, Germany (S-499/2017); Universitätsklinikum Tübingen, Germany (643/2017BO1); Universität zu Lübeck, Germany (17-272); Ludwig-Maximilians-Universität München, Germany (17-640); Ärztekammer Hamburg, Germany (MC-316/17); Technische Universität Dresden, Germany (AK 464122017). All patients or their legal guardians provided written informed consent prior to inclusion.

Note that full information on the approval of the study protocol must also be provided in the manuscript.
